# Pucker sign in proximal humeral fractures: implications on management

**DOI:** 10.1007/s11751-013-0162-y

**Published:** 2013-06-05

**Authors:** Nipun Jindal, Parmanand Gupta, Ravi Kumar Gupta, Amit Kumar, Ankush Jindal

**Affiliations:** Department of Orthopaedics, Government Medical College and Hospital, Sector 32, Chandigarh, India

**Keywords:** Surgical neck humerus fracture, Puckering, Dimpling, Buttonholing, Open reduction

## Abstract

Fracture of the surgical neck of humerus in young patients is a relatively rare injury. We reviewed the available material on the topic and identified puckering at the shoulder in high-energy fracture of the surgical neck as a finding which has been reported infrequently but signifies a need for open reduction. We present a review of the literature on the subject and our similar experience in two young males who had puckering and ecchymosis at the shoulder.

## Introduction

Fracture of surgical neck of humerus accounts for 12.7 % of proximal humeral fractures [1] with majority of cases occurring in elderly as fragility fractures. However, these fractures can also occur in young patients following high-energy trauma. The energy of trauma together with the force generated by surrounding muscles may result in buttonholing of distal fragment through the deltoid muscle causing puckering on the skin. Skin puckering as a sign of proximal humeral fractures has been described as a physical sign in only two reports worldwide [2, 3]. We review the available literature and consider the implications of such a sign on the treatment protocol observed in such injuries. We also present our experience with two cases of fracture of surgical neck of humerus in young males with buttonholing through the deltoid muscle and subsequent skin puckering.

## Case 1

**Fig. 1:**
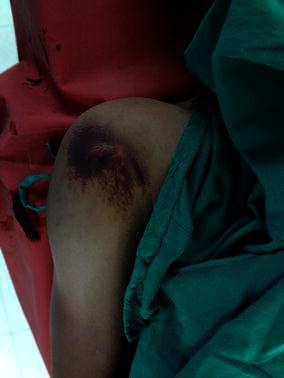
Clinical photograph of Case 1 showing the ecchymosis and puckering at the right shoulder

**Fig. 2:**
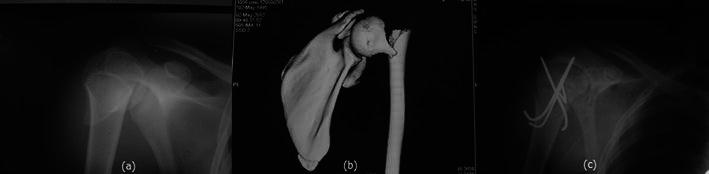
**a** Anteroposterior radiograph revealing the underlying surgical neck humerus fracture with minimal medio-lateral displacement. **b** CT scan image showing angulation with minimal comminution. **c** Postoperative radiograph showing achievement of good reduction with multiple Kirschner wires

A 17 year old male presented with pain and inability to move right shoulder after being involved in a road traffic accident. Physical examination revealed a swollen right shoulder with puckering of skin at the anterior aspect and localised ecchymosis at the puckering site (Fig. 1). Radiographs (Fig. 2a, b) revealed a fracture of surgical neck of humerus (OTA—11-A3.2). Since the skin impingement was causing significant pain to the patient, it was immediately attempted to reduce the fracture to dis-impinge the skin under sedation; however, the attempt failed. An attempt to perform closed reduction and percutaneous pinning was done; the fracture still irreducible under general anaesthesia.

**Fig. 3:**
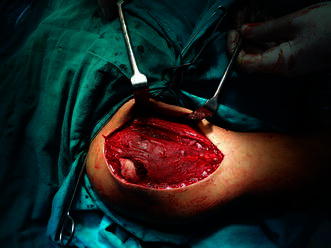
Intraoperative photograph showing buttonholing of humeral shaft through the deltoid

**Fig. 4:**
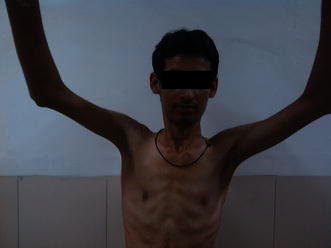
Clinical photograph showing good range of motion at right shoulder after implant removal

Open reduction and internal fixation was then undertaken using deltopectoral approach. The proximal part of the shaft fragment was found to be buttonholed through the deltoid (Fig. 3). The fracture was aligned after reduction of buttonholing and fixed with multiple Kirschner wires (Fig. 2c). Shoulder movements were restricted in a shoulder immobiliser for a period of 4 weeks beyond which short arc pendular exercises were commenced. Gradually, the movements were increased to long arc pendular and circumduction movements and thereafter to wall ladder both side and front. The wires were removed at 6 weeks postoperatively, and full range of motion was achieved at the end of 3 months (Fig. 4).

## Case 2

**Fig. 5:**
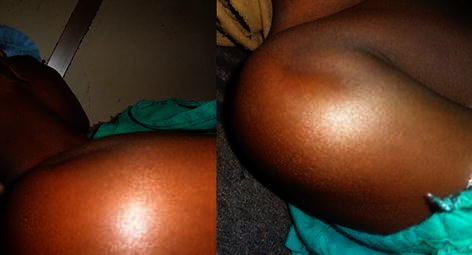
Clinical photograph Case 2 showing puckering at right shoulder

**Fig. 6:**
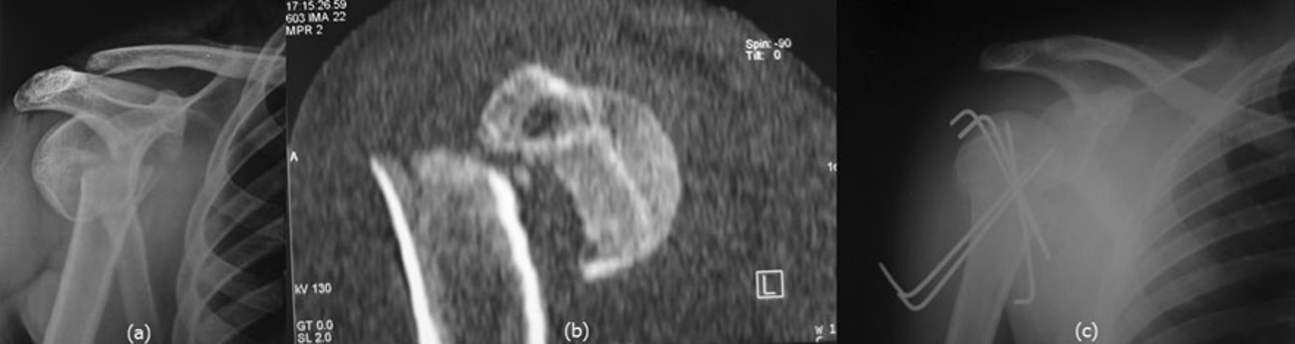
**a** Anteroposterior radiograph showing fracture of surgical neck of humerus. **b** CT scan image showing the fracture pattern with angulation. **c** Postoperative reduction with multiple K wires

**Fig. 7:**
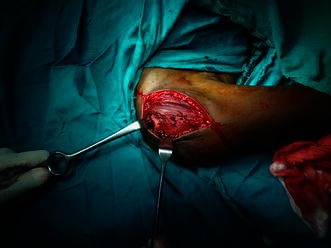
Intraoperative photograph of Case 2 showing buttonholed fragment through the deltoid

A 16 year old boy presented to us with a swollen and painful right shoulder after a fall. The anterior aspect had puckering (Fig. 5) similar to the previous case but no ecchymosis. Radiographs showed a fracture of surgical neck of humerus (Fig. 6a, b). Closed reduction was attempted immediately under sedation and later in general anaesthesia; however, both attempts failed. Open reduction of the buttonholing and internal fixation were done with multiple Kirschner wires (Fig. 6c) using a deltopectoral approach (Fig. 7). The wires were removed at 6 weeks after the surgery. After similar postoperative rehabilitative regimen as followed in the previous case, the patient regained his full shoulder range of motion at 10 weeks.

## Discussion

Type of fracture according to Neer [4] is a major determinant of prognosis in the treatment of proximal humeral fractures. The degree of comminution and fracture fragment separation as we approach the far end of the spectrum signifies a greater amount of energy being transferred at the time of primary impact. However, since the majority of proximal humeral fractures occur in the elderly population with osteopenic bones, the amount of comminution might not point accurately to the energy of the trauma.

Displaced surgical neck fractures have a predominantly unimodal age distribution with maximum cases occurring after age 70. A study by Court-Brown et al. [5] found that only 3.2 % of such fractures occur below the age 40 with all of them attributable to road traffic accidents. The major determinants of treatment of such fractures were age of the patient and the degree of initial displacement. Conservative management of these fractures in elderly usually yields good results with low rates of pseudoarthrosis [6]. The union occurs eventually with a good functional outcome even if malunited in angulation because of a wider range of motion at shoulder. For this reason, non-operative management may be preferred in the elderly [7, 8].

Marked displacement/angulation of fragments and a younger age at involvement suggest an operative course of management. Closed reduction and percutaneous pinning remains the procedure of choice in these cases, closed reduction being achievable in majority of cases [9]. A good bone quality in these patients permits excellent anchorage of Kirschner wires for maintenance of reduction till fracture union. Disruption of surrounding soft tissues leading to anterior angulation and anterior displacement of the distal fragment may result due to the force vector of pectoralis major tendon. Closed reduction usually proceeds in such cases by longitudinal traction along with minimal abduction and some flexion to relax the Pectoralis major fully [10]. However, buttonholing through surrounding muscles like deltoid might render these fractures irreducible. The fragments may come to lay subcutaneously giving rise to pucker marks on the skin. A still higher impact at time of trauma may cause the fragments to cause internal compounding as well. However, the latter may be associated with wider muscle lacerations permitting an easier reduction than the ones with puckering.

The appearance of skin puckering is analogous to the puckering observed in paediatric supracondylar humeral fractures of the extension type with the distal spike of the proximal fragment buttonholing through the brachialis. Brubacher et al. [11] in their review of supracondylar humeral fractures, recognised that presence of skin dimple indicated that the fracture might not reduce by simple manipulation alone. However, a search of literature did not give the exact percentage of cases with positive puckering sign which did require an open reduction.

However, in surgical neck humeral fractures, puckering sign correlated with a failure of closed reduction in all the four cases (Table 1) and thus itself forms an indication of open reduction. We observed that attempts at closed reduction dramatically increased the puckering pointing to a clenching effect of the muscle on the spiked fragment. The sign thus indirectly suggested a necessity of operative plan from the beginning. Puckering can also be approved by preoperative ultrasonic examination of the fractured proximal humerus. Ultrasonographic evaluation has successfully diagnosed, determined the quality of reduction and any soft tissue interposition between fracture fragments in many studies [12, 13], particularly in paediatric fractures. However, a study conducted by Bner et al. [13] found that ultrasound was less accurate for metaphyseal fractures. The role of ultrasound in similar cases of proximal humeral fractures appears more convincing where the buttonholing presents as ecchymosis alone without puckering.

**Table 1:** Summary of the cases of proximal humerus fractures reported till date

Year reported	Author	Age, sex	Mode of trauma	Puckering	Ecchymosis	Fracture type	Management
2008	Alshryda et al.	53, F	Fall from height	+	+	Surgical neck	ORIF
2011	Davarinos et al.	19, F	Fall from height	+	+	Surgical neck; with comminution	ORIF
2013	Jindal et al.	17, M	Road traffic accident	+	+	Surgical neck	ORIF
		16, M	Fall from height	+	–	Surgical neck	ORIF

*ORIF* open reduction and internal fixation

## Conclusion

Pucker sign at the shoulder points to an underlying surgical neck humeral sustained as a result of high-energy trauma. Puckering and ecchymosis at the contour of the shoulder in proximal humeral fractures should be acknowledged as a sign of buttonholing and a hint to failure of closed reduction if pursued. The presence of this sign renders the injury a fracture of necessity.

## References

[1] Court-Brown CM, McQueen MM, Garg A (1999). The epidemiology and outcome of proximal humeral fractures.

[2] Alshryda SJ, Sodak PA (2008). Skin puckering as a sign of humeral neck fracture. Ann RColl Surg Engl.

[3] Davarinos N, Ellanti P, Khan Bhambro KS, Keogh P (2011). Skin puckering an uncommon sign of underlying humeral neck fracture: a case report. Ir J Med Sci.

[4] Neer CS (1975). Four-segment classification of displaced proximal humeral fractures. AAOS Instr Course Lect..

[5] Court-Brown M, Garg A, McQueen MM (2001). The translated two-part fracture of the proximal humerus Epidemiology and outcome in the older patient. J Bone Joint Surg Br.

[6] Maheshwari J, Pandey VK (2012). Pseudoarthrosis of the surgical neck of humerus treated by buttressing with a medial cortico-cancellous graft. Indian J Orthop.

[7] Zyto K, Ahrengart L, Sperber A (1997). Treatment of displaced proximal humeral fractures in elderly patients. J Bone Joint Surg Br.

[8] Michael Robinson C, Bucholz RW, Court-brown CM, Heckman JD, Tornetta P (2006). Proximal humerus fracture. Rockwood and Green’s fracture in adults.

[9] Kocialkowski A, Wallace WA (1990). Closed percutaneous K-wire stabilization for displaced fractures of the surgical neck of the humerus. Injury.

[10] Chen CY, Chao EK, Tu YK, Ueng SW, Shih CH (1998). Closed management and percutaneous fixation of unstable proximal humerus fractures. J Trauma.

[11] Brubacher JW, Dodds SD (2008). Pediatric supracondylar fractures of the distal humerus. Curr Rev Musculoskelet Med.

[12] McManus JG, Morton MJ, Crystal CS, McArthur TJ, Helphenstine JS, Masneri DA, Young SE, Miller MA (2008). Use of ultrasound to assess acute fracture reduction in emergency care settings. Am J Disaster Med.

[13] Bner U, Schlicht W, Outzen S, Barthel M, Halsband H (2000). Ultrasound in the diagnosis of fractures in children. J Bone Joint Surg [Br].

